# Eosinophilic fasciitis (Shulman syndrome), a rare entity and diagnostic challenge, as a manifestation of severe chronic graft-versus-host disease: a case report

**DOI:** 10.1186/s13256-021-02735-3

**Published:** 2021-03-15

**Authors:** Thomas Chalopin, Nicolas Vallet, Marion Morel, Raphael Maguet, Louis d’Alteroche, Gonzague de Pinieux, Olivier Hérault, Emmanuel Gyan, Laurent Sutton, Alban Villate

**Affiliations:** 1 Department of Hematology and Cell Therapy, University Hospital of Tours, 2 Boulevard Tonnellé, 37044 Tours Cedex 9, France; 2 Department of Medical Imaging, University Hospital of Tours, Tours, France; 3 Department of Hepatology, University Hospital of Tours, Tours, France; 4 Department of Pathology, University Hospital of Tours, Tours, France; 5 Department of Biological Hematology, University Hospital of Tours, Tours, France; 6 Groupe Innovation et Ciblage Cellulaire EA 7501, ERL 7001 LNOx, CNRS–University of Tours, Tours, France; 7 Clinical Investigation Center, University Hospital of Tours–INSERM U1415, Tours, France

**Keywords:** Multiple myeloma, Allogenic hematopoietic stem cell transplantation, Eosinophilic fasciitis, Chronic graft-versus-host-disease

## Abstract

**Background:**

Shulman’s disease, or eosinophilic fasciitis (EF), is a rare autoimmune disease, characterized by sclerodermic skin lesions with progressive induration and thickening of the soft tissues. Chronic graft-versus-host-disease (GVHD) presenting as EF is a very rare manifestation of cutaneous GVHD.

**Case presentation:**

We report an unusual case of EF in a 46-year-old Caucasian male patient who had received an allogenic hematopoietic stem cell transplantation in the context of relapsed/refractory multiple myeloma. The diagnosis was challenging, with the patient presenting hepatic dysfunction, normal eosinophils count, and incomplete clinical signs. Magnetic resonance imaging (MRI) and skin biopsy confirmed the diagnosis of EF. Early initiation of specific treatment with corticosteroids and prednisolone achieved complete response.

**Conclusion:**

In practice, incomplete signs in this rare complication should lead to MRI as it is a major tool to guide decision-making based on the skin biopsy, allowing a rapid diagnosis and the initiation of treatment without delay.

## Background

Shulman’s disease, or eosinophilic fasciitis (EF), is a rare autoimmune disease characterized by sclerodermic skin lesions with progressive induration and thickening of the soft tissues [1–3]. Other clinical manifestations can be fever, dyspnea, myalgia, synovitis, and lung lesions [2]. The main biological abnormalities are hypereosinophilia, hypergammaglobulinemia, and high levels of C-reactive protein and aldolase [4].

Chronic graft-versus-host disease (GVHD) is the most frequent late complication of allogenic hematopoietic stem cell transplantation (ASCT) [5]. Skin is the most common organ affected (75%), with presentation of a large variety of lesions [6]. Chronic GVHD (cGVHD) presenting as EF is an uncommon manifestation of cutaneous GVHD, with an incidence of 0.5–6% [6, 7]. The final diagnosis is based on immunohistopathology of a skin-to-muscle biopsy [3, 8]. The mechanisms of the pathogenesis of EF syndrome are still unclear, but may involve the products of eosinophil granules, such as neurotoxins, collagen, cytokines, or chemokines, as well as CD4^+^ Th1 and Th17 T-cell polarization [9].

Here, we report an uncommon case of EF, without hypereosinophilia, as a complication of cGVHD 7 months after a familial sibling ASCT for refractory multiple myeloma.

Written informed consent was obtained from the patient for publication of this case report and any accompanying images.

## Case presentation

We report an unusual case of EF in a 46-year-old Caucasian male patient who received an ASCT in the context of relapsed/refractory multiple myeloma. He was a farmer and had no reported personal and familial medical history of note. The diagnosis was made in November 2013, with the patient presenting kappa IgG gammopathy (44 g/L), 41% medullar plasma cell infiltration, bone lesions, and hypercalcemia (2.76 mmol/L). The Revised International Staging System (R-ISS) score was 2 (ISS 2, normal karyotype, elevated lactate dehydrogenase at 450 IU/L). Frontline therapy was four courses of bortezomib with thalidomide and dexamethasone (VTD), followed by ASCT in February 2014, then two cycles of consolidation with VTD. Twenty months after transplantation, the appearance of bone lesions on positron emission tomography-computed tomography (PET-CT) imaging with ^18^fluorodeoxyglucose led to the diagnosis of relapse. Second-line treatment began in March 2016 with lenalidomide and dexamethasone. In June 2017, a second diffuse bone relapse was revealed by PET-CT imaging, which required radiation therapy of the right pelvis, followed by treatment with daratumumab, bortezomib, and dexamethasone. One year later, the patient showed a partial response according to International Myeloma Working Group criteria [10].

Given the young age of the patient and the high risk of progression, an ASCT from an HLA-identical sibling donor (sister) was planned. The donor/recipient status was as follows: O+/O+, CMV−/−, EBV +/+, toxoplasmosis +/+, and hepatitis B virus −/−. Myeloablative conditioning with high-dose melphalan (140 mg/m^2^) and total body irradiation (8 Gy) was performed in February 2019. Prophylaxis of acute GVHD included cyclosporin, methotrexate (days + 1, 3, 6, and 11) and T-cell depletion with anti-thymocyte globulin (days + 3, + 2). The recovery of hematopoiesis was normal (13 days of aplasia) and no acute GVHD occurred. Three months post-ASCT, minimal extramedullary residual disease was negative on PET-CT imaging, with complete response on electrophoresis and immunofixation. Blood chimerism was 100% donor. Cyclosporin was gradually tapered and discontinued 4 months post-transplant (day + 133).

**Fig. 1.**
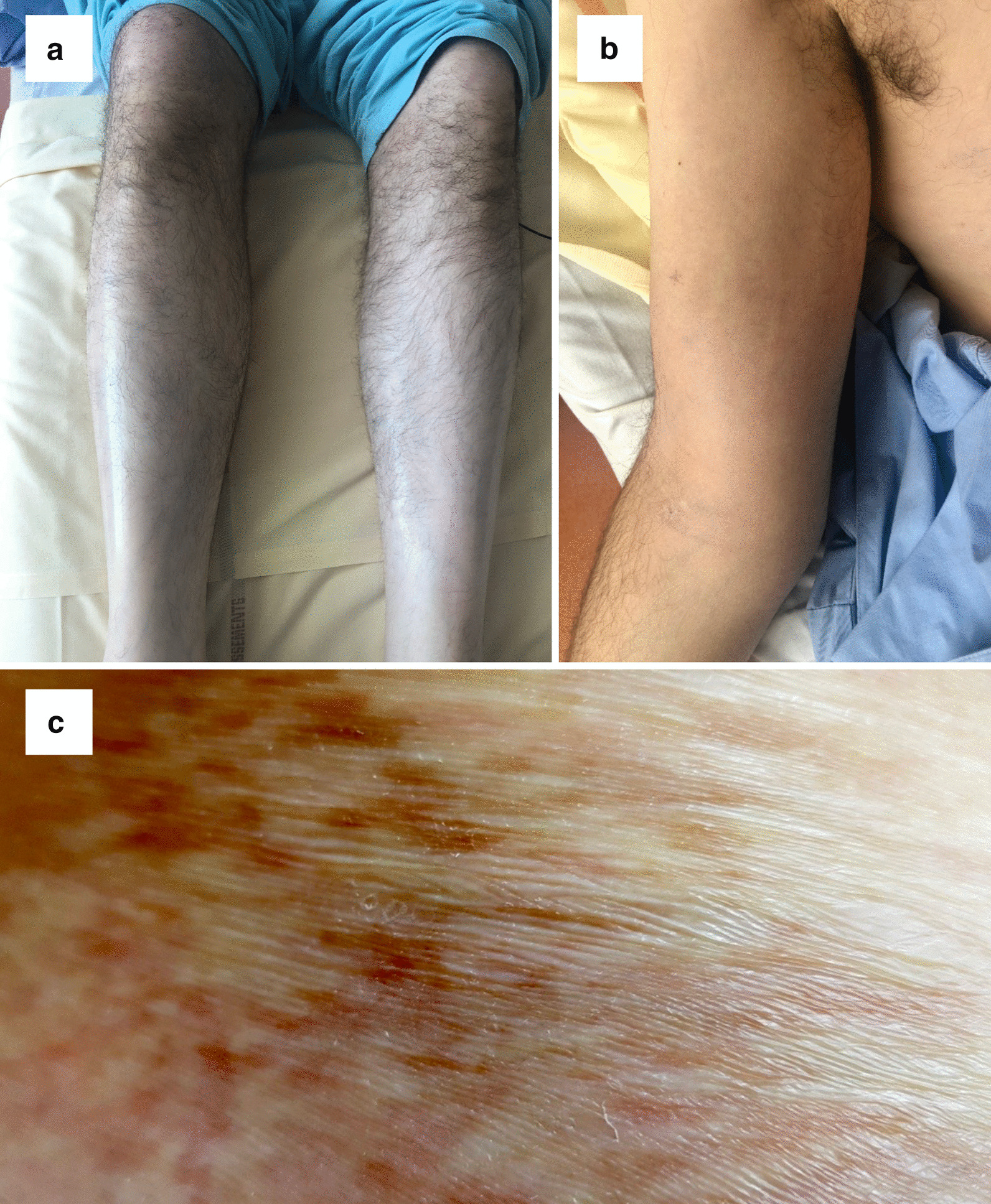
Physical examination of patient. **a** Patient’s legs with bilateral edema, **b** patient’s right arm with the beginning of skin induration and edema, **c** morphea-like lesions on the left thigh

Major hepatic dysfunction with cytolysis and cholestasis occurred 7 months post-transplant (day + 218). The standard etiological workup, including drug-related cause, hepatitis virus serologies and PCR, and autoantibody determination, was negative. Between days + 228 and + 242, he was admitted due to the appearance of edema and pain in the extremities. Physical examination revealed diffuse skin induration, predominant in the limbs, and mucous lichen associated with rare morphoea-like lesions (Fig. 1). The patient was febrile (38.7 °C) with normal blood pressure and the heart pulse was 100 beats/min. We observed dyspnea and global weakness of the limbs, without arthralgia or typical groove sign. Results of a neurological examination were normal.

Results from laboratory studies were as follows: platelets 52 × 10^9^/L, hemoglobin 131 g/L, and leucocytes 4.4 × 10^9^/L, with a normal eosinophil granulocyte count (0.26 × 10^9^/L). Other results included: aspartate aminotransferase 418 UI/L, alanine aminotransferase 302 UI/L, alkaline phosphatase 270 UI/L, gamma-glutamyltransferase 189 UI/L, C-reactive protein 16.5 mg/L, and a low level of albumin at 22 g/L. Specific studies showed positive homogeneous speckled antinuclear antibodies, elevated aldolase levels, and hypergammaglobulinemia. The infection workup was negative.

**Figure 2.**
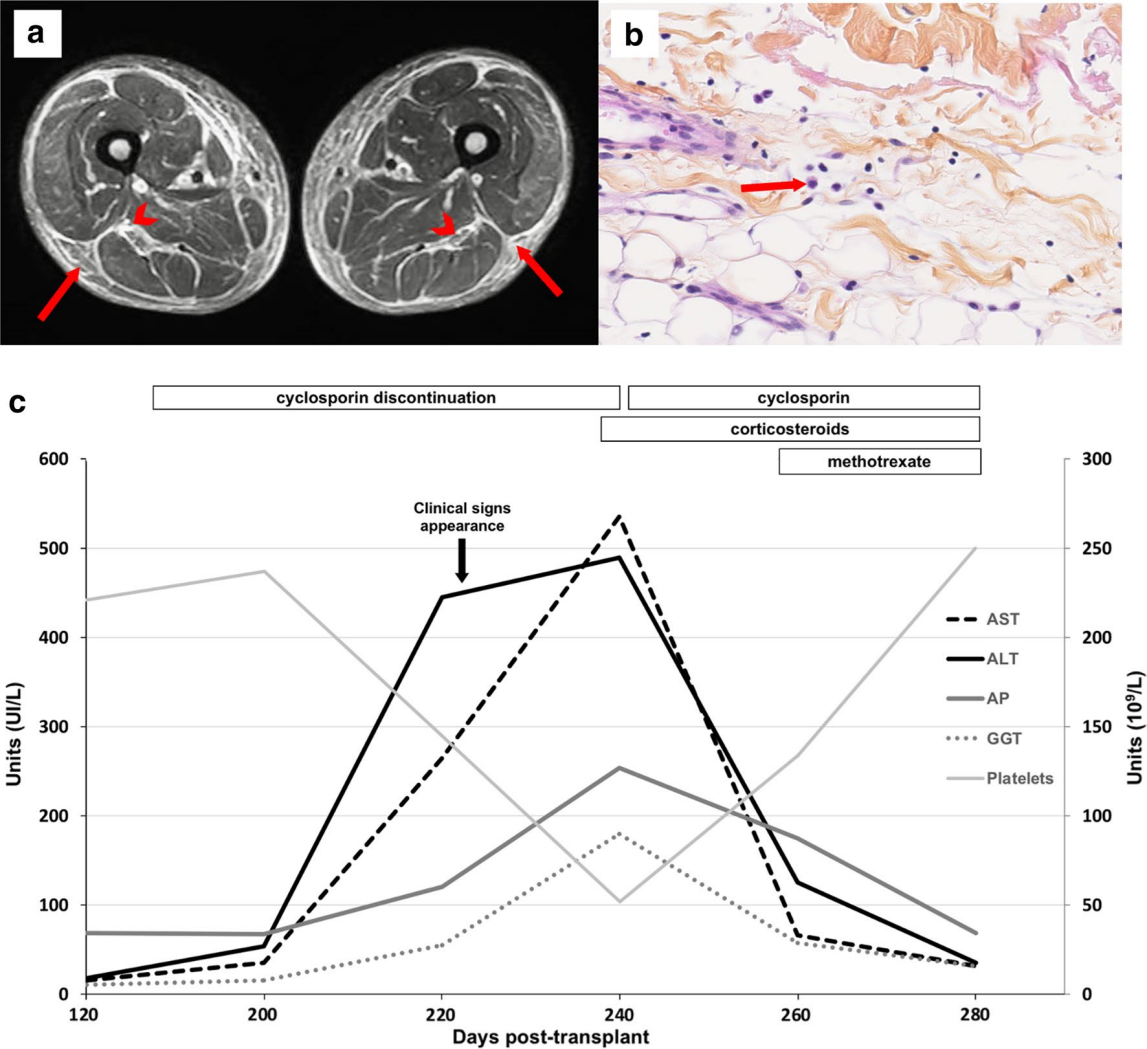
**a** Muscle magnetic resonance image with STIR sequence in the axial plane. The image shows symmetric thickening and high signal intensity in the peripheral deep fasciae (arrows) and intermuscular fasciae (arrowheads), mainly in the posterior muscle compartment of the thigh. **b** Deep skin biopsy (magnification 40×) showing thickening of the fascia due to lymphocyte and plasma cell infiltration. We observed only one eosinophil granulocyte (arrow). **c** Trends in hepatic enzyme elevation and standardization, with platelet counts, during the course of this case of Shulman disease.* AST* Aspartate aminotransferase,* ALT* alanine aminotransferase,* AP* alkaline phosphatase,* GGT* gamma-glutamyltransferase

Total body magnetic resonance imaging showed increased signal intensity in short tau inversion recovery (STIR) sequences in the superficial and deep fasciae (Fig. 2a). Active fasciitis involved all muscles and was predominant in the inferior limbs. No myositis or arthritis was found. A skin-to-muscle biopsy of the left thigh confirmed fasciitis and edema, with rare eosinophil infiltration (Fig. 2b). The fascia was dissociated by loose and slightly inflammatory fibrosis, with edema, fibrin, and fibroblasts. Liver biopsy confirmed concomitant cGVHD, with fibrosis and inflammatory infiltrate featuring lymphocytes and eosinophil granulocytes, without apoptotic bodies.

Corticosteroids (prednisolone, 0.75 mg/kg/day) and cyclosporin (3 mg/kg/day) were initiated, with a good response. Following the standardization of hepatic function, weekly administration of methotrexate (15 mg/m^2^) improved the clinical and biological response (Fig. 2c). Skin induration and edema vanished within a few days. The last follow-up was in October 2020 (13 months later), with sustained complete response and few signs of cGVHD-like oral lichen planus.

## Discussion

Here, we report an interesting case of EF, without hypereosinophilia, as a complication of cGVHD 7 months after a familial sibling ASCT for refractory multiple myeloma. Other signs of cGVHD included significant hepatic dysfunction, confirmed by liver biopsy.

The incidence of EF in patients who have undergone ASCT presenting as cGVHD is rare [6, 7]. To our knowledge, only one case of patient with multiple myeloma has been reported [7]. Eosinophilia is found in 60–90% of cases but is not required for diagnosis [4]. Skin involvement is reported in 90% of cases, with an evolutive natural history (edema, “peau d’orange” appearance, morphea lesions, groove sign). Our patient had similar clinical manifestations as the other cases, presenting with pain, swelling, induration, and edema. In our case, biological abnormalities with hepatic dysfunction preceded the appearance of clinical signs. Associated organ dysfunction is also possible, with a predominance of other skin symptoms with mouth or sicca involvement as signs of GVHD [7]. Liver involvement remains uncommon, and standard workup must be done to rule out other possible causes.

MRI is an important diagnostic and prognostic tool of EF, typically showing increased signal intensity within the fascia [11, 12]. It is the ideal imaging modality both for selecting the best site for biopsy and monitoring the course of EF following initiation of treatment. In our case, MRI was crucial in enabling an early diagnosis in combination with other data (clinical, biological and anatomical pathology). Although not strictly required for diagnosis, MRI results appear to be required as part of the initial assessment of EF.

There are no consensual recommendations for EF treatment, and the cornerstone of treatment remains corticosteroids, with an initial dose of 0.5–1 mg/kg per day [2, 3, 13]. The duration of therapy is highly variable, with a good response in most cases. In steroid-refractory patients, the addition of immunosuppressive drugs to the therapeutic regimen, such as cyclosporin or methotrexate, is useful [3, 14, 15]. Other strategies, such as extracorporeal photopheresis, tyrosine kinase inhibitors, or sirolimus may be required [7, 16]. Lebeaux *et al*. showed that the response is worse if the interval from diagnosis is longer than 6 months (odds ratio 15) [3]. In reported cases, only a few patients had complete response with the disappearance of skin signs and improved laboratory paramenters [7]. In our case, we initiated treatment with cyclosporin and prednisolone early, 15 days after the first appearance of the clinical symptoms. The patient’s response to this treatment was complete, with the recovery of hepatic function and a complete resolution of skin lesions.

## Conclusion

We report an unusual case of Shulman syndrome presenting as cGHVD, without hypereosinophilia, and with complete response upon early initiation of treatment. Incomplete signs in this rare complication should lead to MRI as it is a major tool to guide the skin biopsy, allowing a rapid diagnosis and the initiation of treatment without delay.

## Data Availability

Not applicable.
